# Porcine Colostrum Protects the IPEC-J2 Cells and Piglet Colon Epithelium against *Clostridioides* (syn. *Clostridium*) *difficile* Toxin-Induced Effects

**DOI:** 10.3390/microorganisms8010142

**Published:** 2020-01-20

**Authors:** Łukasz Grześkowiak, Robert Pieper, Susan Kröger, Beatriz Martínez-Vallespín, Anja E. Hauser, Raluca Niesner, Wilfried Vahjen, Jürgen Zentek

**Affiliations:** 1 Institute of Animal Nutrition, Freie Universität Berlin, 14195 Berlin, Germany; 2 German Rheumatism Research Center, Berlin, A Leibniz Institute, 10117 Berlin, Germany; 3 Intravital Microscopy and Immune Dynamics, Charité–Universitätsmedizin Berlin, 10117 Berlin, Germany; 4 Dynamic and Functional in vivo Imaging, Veterinary Medicine, Freie Universität Berlin, 14163 Berlin, Germany

**Keywords:** *Clostridium*, *Clostridioides difficile*, IPEC-J2, Ussing chamber, toxins, colostrum, milk, piglet, cytoskeleton, tight junction proteins, immunofluorescence

## Abstract

*Clostridioides difficile* toxins are one of the main causative agents for the clinical symptoms observed during *C. difficile* infection in piglets. Porcine milk has been shown to strengthen the epithelial barrier function in the piglet’s intestine and may have the potential to neutralise clostridial toxins. We hypothesised that porcine colostrum exerts protective effects against those toxins in the IPEC-J2 cells and in the colon epithelium of healthy piglets. The IPEC-J2 cells were treated with either the toxins or porcine colostrum or their combination. Analyses included measurement of trans-epithelial electrical resistance (TEER), cell viability using propidium iodide by flow cytometry, gene expression of tight junction (TJ) proteins and immune markers, immunofluorescence (IF) histology of the cytoskeleton and a TJ protein assessment. Colon tissue explants from one- and two-week-old suckling piglets and from five-week-old weaned piglets were treated with *C. difficile* toxins in Ussing chamber assays to assess the permeability to macromolecules (FITC-dextran, HRP), followed by analysis of gene expression of TJ proteins and immune markers. Toxins decreased viability and integrity of IPEC-J2 cells in a time-dependent manner. Porcine colostrum exerted a protective effect against toxins as indicated by TEER and IF in IPEC-J2 cells. Toxins tended to increase paracellular permeability to macromolecules in colon tissues of two-week-old piglets and downregulated gene expression of occludin in colon tissues of five-week-old piglets (*p* = 0.05). Porcine milk including colostrum, besides other maternal factors, may be one of the important determinants of early immune programming towards protection from *C. difficile* infections in the offspring.

## 1. Introduction

*Clostridioides* (syn. *Clostridium*) *difficile* has been documented as an important cause of uncontrolled enteritis outbreaks in neonatal pigs [1,2]. Toxins A (TcdA) and B (TcdB) besides a binary toxin are the main infection agents leading to loss of epithelial integrity, immune response and intestinal damage [3]. Their action is related to the modulation of the intestinal epithelial cell physiology and disruption of barrier function via inactivation of Rho proteins involved in the formation of the cytoskeleton. This leads to disruption of tight junction (TJ) proteins and finally to a loss of epithelial integrity, as demonstrated in porcine and human cell lines [4,5,6,7]. Such a scenario of events may lead to the typical signs of *C. difficile* infection (CDI) [8,9] with severe consequences, such as animal death or growth retardation in piglets that survived the infection [10]. However, why some piglets are asymptomatic carriers of high concentrations of *C. difficile* spores and toxins and others are not, is not exactly clear [11]. In addition, the reasons why some piglets of the same litter get sick and others do not remain unknown so far.

Maternal factors may play an important role in protection from CDI. Porcine milk is rich in bioactive components and has been shown to be beneficial for the offspring [12,13]. Porcine milk has also been demonstrated to strengthen the epithelial barrier function in the piglet gut [14] and previous evidence suggests that it could have the potential to neutralise clostridial toxins [8] and possibly prevent CDI.

Based on the association between the mother sow and offspring in relation to piglet susceptibility to CDI, we hypothesised that porcine colostrum exerts protective effects against *C. difficile* toxins on IPEC-J2 cells in vitro and in colon epithelium of healthy suckling and weaned piglets. We therefore treated IPEC-J2 cells and the explants of colon tissues with a *C. difficile* culture supernatant containing toxins. In addition, IPEC-J2 cells were treated with porcine colostrum alone and together with the toxins. Next, we assessed the signs of intoxication through measurement of cell trans-epithelial electrical resistance (TEER) and cell viability, colon epithelium permeability to macromolecules, gene expression of TJ proteins, as well as the immunofluorescence (IF) analysis of the cytoskeletal structure and TJ proteins in IPEC-J2 cells.

## 2. Materials and Methods

### 2.1. Ethical Statement

The institutional and national guidelines for the care and use of animals were followed and the study was approved by the State Office of Health and Social Affairs (“Landesamt für Gesundheit und Soziales Berlin”, LAGeSo Reg G0255/14 (20 January 2015) and G0269/16 (16 February 2017).

### 2.2. C. difficile Toxins

To obtain the spent culture supernatant with toxins, spores of infectious *C. difficile* ribotype 078, known to produce toxins A and B and carrying genes encoding a binary toxin, were inoculated into BHI media supplemented with yeast extract and taurocholate and without L-cysteine [8,15,16,17]. They were then incubated at 37 °C for 1–2 weeks to produce a sufficient concentration of toxins. Thereafter, the spent culture supernatant was centrifuged at 10,000× *g* for 10 min, filtered through 0.22 µm pore size MF-Millipore membrane filters (Merck, Darmstadt, Germany) and subjected to a commercial ELISA kit (tgcBIOMICS GmbH, Bingen, Germany) to determine the concentrations of TcdA and TcdB [11]. Previously performed tests showed that a two-hour incubation at a toxin concentration of 13 ng/mL of TcdA and 8 ng/mL of TcdB was suitable to demonstrate detrimental changes in IPEC-J2 cells by lowering the TEER values within time but without inducing prominent cell damage or death (light microscopic evaluation), so that further manipulations such as cell washing could be performed without loss of the cells. A one-fold higher concentration of the toxins or a longer incubation time led to a drastic fall in TEER [5], as well as disintegration and break-up of the cells, which could be seen under a light microscope. In addition, previous preliminary tests showed that slight variations of the established toxin concentration had only a small impact on the TEER values. Therefore, further experiments using colon tissues and IPEC-J2 cells were performed with the toxins at a concentration range of 5.4–17 ng/mL for TcdA and 1.6–8 ng for TcdB. Further sample cell collection for the expression analysis of certain genes, as well as the staining of the cytoskeleton and TJ proteins in IPEC-J2 were performed also two hours after cell treatment with the toxins.

### 2.3. Porcine Colostrum

The late colostrum was obtained from four lactating sows one day post-partum and stored frozen at −30 °C until further analyses. The presence of IgG anti-toxin A antibodies was previously confirmed by immunoassay [5].

### 2.4. IPEC-J2 Cell Culture Conditions

IPEC-J2 cells (ACC 701) obtained from the DSMZ (German Collection of Microorganisms and Cell Cultures, Braunschweig, Germany) and stored at our institute, were grown in plastic flasks (Greiner Bio-One International GmbH, Kremsmünster, Austria) at 37 °C in an atmosphere of 5% CO_2_, and maintained in Dulbecco’s MEM/Ham’s F-12 (DMEM) (Biochrom GmbH, Berlin, Germany) supplemented with 5% foetal calf serum, 100 units penicillin/mL and 100 mg streptomycin/mL (Biochrom GmbH, Berlin, Germany), as previously described [18]. The cells from passage 43 to 62 were used for the experiments. They were seeded to a density of 5 × 10^5^ cells/well except for IF, which was 10^5^ cells/well. IPEC-J2 cells were maintained in the cell culture plates during 7 days (except for IF, 5 days) at 37 °C with 5% CO_2_ changing to a fresh medium every second day, until they reached confluence, using the value of the transepithelial electrical resistance (TEER) as an indicator [19]. Once confluence was achieved, the cells were subjected to the experiments. All experiments involving IPEC-J2 cells were determined in three independent experiments and each assay was performed in triplicate.

### 2.5. Treatment of IPEC-J2 Cells with Toxins and Porcine Colostrum

The spent culture supernatant containing toxins was pre-diluted in DMEM. The confluent cells were treated with the following: (1) DMEM growth medium as a control; (2) porcine colostrum (final dilution in DMEM was 4×); (3) spent culture supernatant containing toxins; and (4) porcine colostrum + spent culture supernatant containing toxins (at a concentration range of 5.4–17 ng/mL for TcdA and 1.6–8 ng for TcdB), both applied at the same time onto the cells.

### 2.6. TEER Measurement of IPEC-J2 Cells

For the TEER assay, IPEC-J2 cells were transferred to ThinCertTM cell culture inserts (polyethylene terephthalate capillary pore membranes; 0.4 µm pore size; Greiner BioOne, Frickenhausen, Germany) compatible with 6-well plates and treated as described above. The TEER was measured every hour until five hours and at 24 h post-treatment. Measurement was carried out using an epithelial Voltammeter EVOM^2^ with a chopstick electrode STX2 (World Precision Instruments Inc., Sarasota, FL, USA). The data were expressed as the percentage of TEER before incubation.

### 2.7. Immunofluorescence Microscopy of IPEC-J2 Cells

After treatment with the toxins and porcine colostrum, the cells were washed in PBS, fixed in 1% methanol-free formaldehyde for 30 min, permeabilised using 0.1% Triton X-100 for 10 min and blocked in 5% foetal calf serum for 30 min. The F-actin rhodamine phalloidin probe and zonula occludens 1 (ZO-1) rat monoclonal antibody were applied on the coverslips containing IPEC-J2 cells and incubated for two hours at room temperature. The cells were then washed in PBS and stained with goat anti-rat IgG Alexa Fluor 594 to detect the primary antibody against ZO-1. The nuclear DNA was stained with DAPI. Fluorescence signals from F-actin and ZO-1 were visualised using a fluorescent confocal microscope (Zeiss LSM 710, Jena, Germany) equipped with a 20x dry objective lens, NA 0.8 and allowing spectral resolution of up to five stainings in parallel, in the range 405 nm to 633 nm. The ZO-1 primary antibody was purchased from Santa Cruz Biotechnology (Heidelberg, Germany). The F-actin probe and goat anti-rat secondary antibodies, DAPI and methanol-free formaldehyde were purchased from ThermoFisher Scientific, Invitrogen™ (Darmstadt, Germany).

### 2.8. IPEC-J2 Cell Viability

To assess the impact of toxins on viability, IPEC-J2 cells were incubated with three different concentrations of toxins, i.e., 13 ng/mL TcdA + 5 ng/mL TcdB, 130 ng/mL TcdA + 50 ng/mL TcdB and 1300 ng/mL TcdA + 500 ng/mL TcdB. After 2 h of incubation, the cells were washed twice in PBS, trypsinisated, stained with propidium iodide and subjected to flow-cytometry analysis (MACSQuant^®^ Analyze, Miltenyi Biotec, Bergisch Gladbach, Germany). The data were expressed as the percentage of dead cells from the total number of cells.

### 2.9. Animals

A total of 12 healthy piglets (German Landrace) housed with their littermates were euthanised at the age of one, two and five weeks (four piglets from each age group). One- and two-week-old piglets were in their suckling period and five-week-old animals were one week after weaning. The piglets were sedated with 20 mg/kg BW of ketamine hydrochloride Ursotamin^®^; Serumwerk Bernburg AG, Germany) and 2 mg/kg BW of azaperone (Stresnil^®^; Jansen-Cilag, Neuss, Germany) and euthanised by intracardial injection of 10 mg/kg BW of tetracaine hydrochloride, mebezonium iodide and embutramide (T61^®^; Intervet, Unterschleißheim, Germany). Following euthanasia, the gastrointestinal tract was removed, and tissue of the proximal colon was sampled and immediately used for electrophysiological measurements.

### 2.10. Ussing Chamber Assay

For determining colonic permeability to macromolecules in the presence of clostridial toxins, the tissues were subjected to Ussing chamber assays, as described previously [20,21]. Briefly, the colon epithelium was stripped of the serosal and muscle layers and mounted in Ussing chambers (three chambers/piglet/treatment) and bathed in 38 °C modified Krebs–Ringer buffer solution. After 30 min of condition adjustment in the Ussing chambers, the spent culture supernatant with toxins (final toxin concentration: 13 ng/mL of TcdA and 8 ng/mL of TcdB) was added to the mucosal side of the chambers. Permeability to macromolecules was measured using horseradish peroxidase (HRP; 44,000 Da) and fluorescein isothiocyanate–dextran (FITC-D; 4000 Da). Briefly, the tracer was added to the mucosal side of two chambers per piglet. Two chambers served as controls. Samples were taken from the serosal side immediately before addition and after 15 min. Carbachol was added at the end of the incubation to ensure viability of the tissue. Thereafter, the tissues were unmounted from the Ussing chambers and stored frozen at −80 °C for further gene expression analysis. The HRP activity was measured using the QuantaBlu™ Fluorogenic Peroxidase Substrate Kit (ThermoFischer Scientific, Darmstadt, Germany). The FITC-D fluorescence was measured using a Stratagene MX3000p (Agilent Technologies, Waldbronn, Germany). Measurements of serial dilutions of the tracer molecules were used to convert the results in nanograms per millilitre and micrograms per millilitre for HRP and FITC-D, respectively.

### 2.11. Gene Expression in IPEC-J2 Cells and in Colon Tissues

After treatment, the cells were washed in PBS and re-suspended in RNA stabilisation reagent (RNAlater; Qiagen GmbH, Hilden, Germany). They were then collected by cell scraper, transferred to 1.5 mL tubes and stored frozen at −30 °C until further analyses.

Total RNA from frozen colon tissues (−80 °C) collected in the experiments with Ussing chambers and from IPEC-J2 were extracted using NucleoSpin RNA II kit (Macherey–Nagel GmbH and Company KG, Düren, Germany) according to the manufacturer instructions. The RNA quality and quantity were determined with the Agilent RNA 6000 Nano Kit in an Agilent 2100 Bioanalyzer (Agilent Technologies, Waldbronn, Germany). Transcription into cDNA was performed using the SuperScript III Reverse Transcriptase First-Strand complementary DNA Synthesis System (Invitrogen) in a Sure Cycler 8800 (Agilent Technologies, Waldbronn, Germany). Quantitative real-time PCR (qPCR) was performed using the Brilliant II SYBR Green QPCR Master Mix with Low ROX (Agilent Technologies, Waldbronn, Germany) on a Stratagene MX3000p (Agilent Technologies, Waldbronn, Germany). The expression of the following genes was assessed: claudin-2 (*CLDN-2*), occludin (*OCLN*), ZO-1, mucin-2 (*MUC-2*), interferon-γ (*IFN-γ*), transforming-growth-factor-β (*TGF-β*), tumour-necrosis-factor-α (*TNF-α*), interleukin-10 (*IL-10*) and interleukin-6 (*IL-6*) in colon tissues, and claudin-1 (*CLDN-1*), claudin-3 (*CLDN-3*), claudin-4 (*CLDN-4*), *OCLN*, ZO-1 and TGF-β in IPEC-J2 cells. The 60S ribosomal protein L13 (*RPL13*), succinate dehydrogenase subunit A (*SDHA*) and β2-microglobulin (*β2-glob*) were selected as housekeeping genes and used for data normalisation for both colon tissues and IPEC-J2 cells. Primer sequences and annealing temperatures of the assessed genes were taken from earlier studies [21,22].

### 2.12. Statistics

Values of TEER (%) and cell viability (%) were not normally distributed and thus analysed by Kruskal–Wallis H and Mann–Whitney U tests where applicable. The gene expression data of colon tissues and IPEC-J2 cells were analysed by REST software (Qiagen GmbH, Munich, Germany) [23]. In the REST gene expression analysis, IPEC-J2 cells treated with porcine colostrum were considered as the control group to which other treatments were compared. Differences between treated and control piglets for FITC-dextran and HRP at different age groups were analysed by Mann–Whitney U tests. *p*-values equal or below 0.05 were considered significant. SPSS version 24.0.0.0 (IBM, SPSS Statistics, Chicago, IL, USA) was used to calculate statistical differences.

## 3. Results

### 3.1. Porcine Colostrum Improves the Integrity of IPEC-J2 Cells and Protects from Toxin-Induced Effects

**Figure 1:**
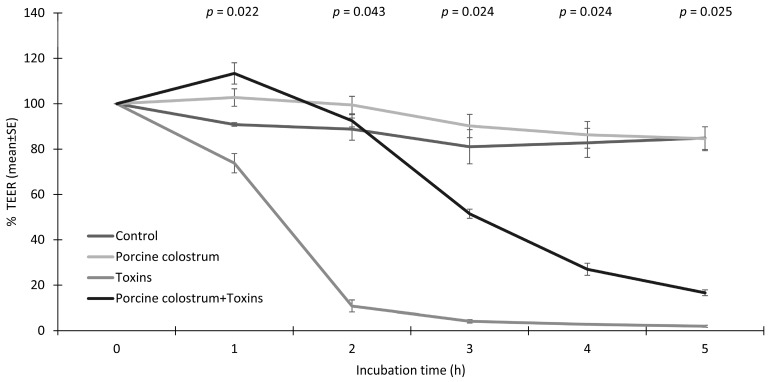
Percentage of transepithelial electrical resistance (TEER), as compared to the TEER before treatment, of IPEC-J2 cells treated with growth medium (Control), porcine colostrum, *C. difficile* toxins (Toxins) and a porcine colostrum and toxin complex (Porcine colostrum + Toxins). The data summarises three independent experiments, each performed in triplicate. The Kruskal–Wallis H test was used to test differences between the groups (significance *p* ≤ 0.05).

TEER development of IPEC-J2 cells treated with porcine colostrum and toxins is shown in Figure 1. As expected, toxins decreased the trans-epithelial resistance of IPEC-J2 cells in a time-dependent manner. On the other hand, porcine colostrum exerted a protective effect on cell integrity against toxins. The integrity of IPEC-J2 cells after two hours of incubation was 88.8% in the control medium, 99.5% in the porcine colostrum, 10.83% in the toxins and 92.5% in the porcine colostrum + toxin complex (*p* = 0.043) of the initial TEER values before treatment. The protective effect of porcine colostrum against toxin-induced events in IPEC-J2 cells was revealed during the first five hours of TEER measurements. The incubation of IPEC-J2 cells in the porcine colostrum vs. control medium led to a slight improvement in the cell integrity during the first five hours of measurement. After 24 h of incubation the integrity of the IPEC-J2 was 90.2% ± 2.04% in the control medium, 41.3% ± 1.96% in the porcine colostrum, 0.83% ± 0.37% in the toxins and 1.73% ± 0.41% when incubated in the porcine colostrum + toxin complex (*p* = 0.083) of the initial TEER values.

**Figure 2:**
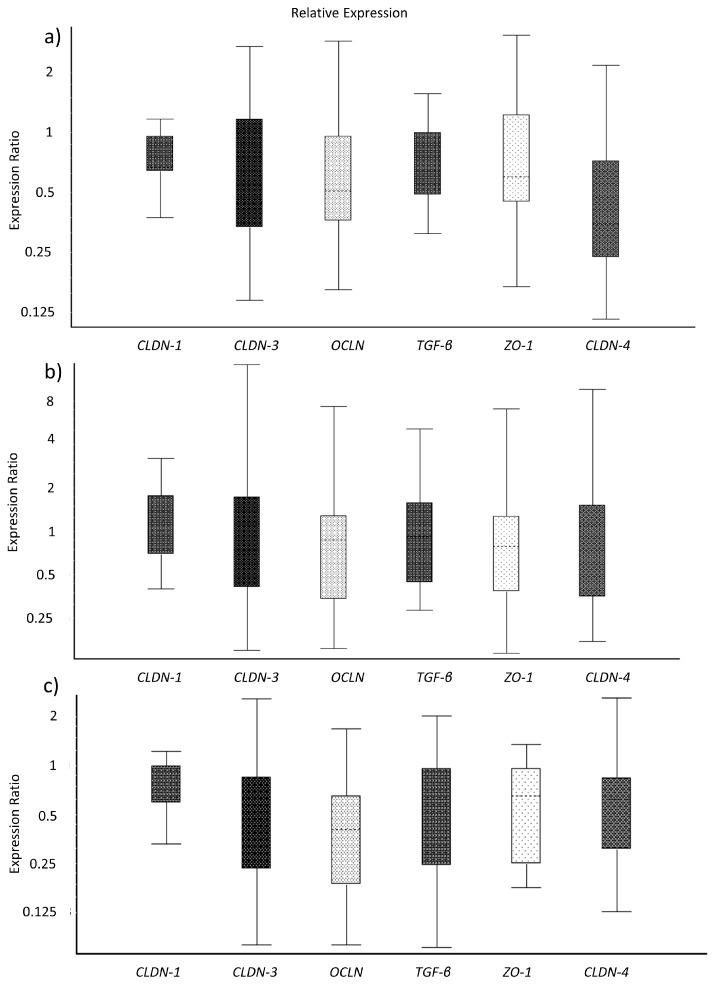
Gene expression ratios of tight junction proteins and immune markers in IPEC-J2 treated with growth medium (ratio Control/Porcine colostrum) (**a**), *C. difficile* toxins (ratio Toxins/Porcine colostrum) (**b**), and a porcine colostrum and toxin complex (ratio Porcine colostrum + Toxins/Porcine colostrum) (**c**), analysed after two hours of incubation. The gene expression data were analysed by REST software.

The analyses of gene expression of TJ proteins and immune markers of IPEC-J2 cells did not reveal significant differences between the treated cells (Figure 2a–c). However, the expression ratio of the cells incubated in the porcine colostrum + toxin complex and in the control medium were numerically lower as compared to incubation in porcine colostrum alone.

### 3.2. Porcine Colostrum Protects Cell Structures against C. difficile Toxins

**Figure 3:**
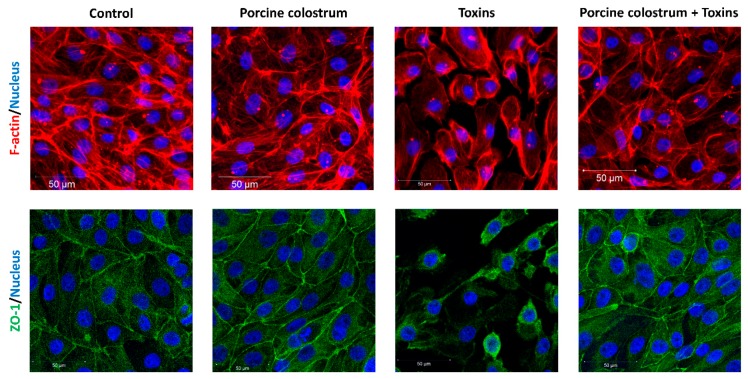
IPEC-J2 cells treated with growth medium (Control), porcine colostrum, *C. difficile* toxins (Toxins) and a porcine colostrum and toxin complex (Porcine colostrum + Toxins). Immunofluorescence confocal microscopic visualisation (M-20X) of F-actin cytoskeleton (red), ZO-1 tight junction proteins (green) and nuclear DNA (blue). Incubation in growth medium or porcine colostrum does not affect the F-actin or ZO-1 in cells. Treatment with *C. difficile* toxins reduces the amount of F-actin and ZO-1 in cells. Toxins also reduce the number of cells as shown by DAPI staining. By combining porcine colostrum with toxins, an improvement of the F-actin cytoskeleton and ZO-1 TJ protein is observed, as compared to the effect of toxins alone.

Neither control growth medium nor porcine colostrum caused a detrimental effect of F-actin or ZO-1 in IPEC-J2 cells, as revealed by IF analysis (Figure 3). On the other hand, the cells treated with the toxins demonstrated drastic structural changes of the F-actin cytoskeleton and TJ protein ZO-1, leading to deformation and disappearance of the cell structures, including shrinking of the nucleus. The contact between the cells in toxin-treated wells was clearly impaired, indicating decreased barrier function. The F-actin and ZO-1 of the cells treated with the porcine colostrum + toxin complex remained largely intact, indicating a protective effect of porcine colostrum against toxins on the IPEC-J2 cells. The cell number remained the same as indicated by DAPI staining.

### 3.3. Toxins Reduce IPEC-J2 Cell Viability and Increase Permeability of Colon Epithelia

**Figure 4:**
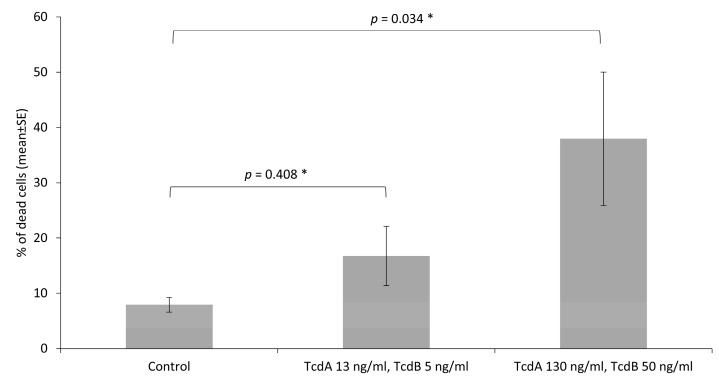
Percentage of dead IPEC-J2 cells as compared to the total number of cells, measured after two-hour incubation in a growth medium (Control) and *C. difficile* toxins at two different concentrations (13 ng/mL + 5 ng/mL and 130 ng/mL + 50 ng/mL for TcdA and TcdB, respectively) resulting from the flow cytometry analysis of propidium iodide staining. The data summarises three independent experiments, each performed in triplicate. The Kruskal–Wallis H test was used to test overall difference between the groups, *p* = 0.039. * The Mann–Whitney U adjusted post-hoc test was used to test differences between the control and treatment groups (significance *p ≤ 0.05*).

The number of dead cells was determined by flow cytometry analysis of propidium iodide staining. Toxins at a concentration of 13 ng/mL (TcdA) + 5 ng/mL (TcdB) and 130 ng/mL (TcdA) + 50 ng/mL (TcdB) increased numerically the percentage of dead IPEC-J2, as compared to non-treated cells (*p* = 0.408 and *p* = 0.034, respectively) (Figure 4). The highest applied concentration of toxins (1300 ng/mL of TcdA and 500 ng/mL of TcdB) led to an inadequate lower percentage of stained cells with propidium iodide, as compared to the cells treated with the two lower concentrations of toxins, which could be due to a massive detachment of the dead cells from the plate bottom wells, and consequently its loss during the washing steps.

**Figure 5:**
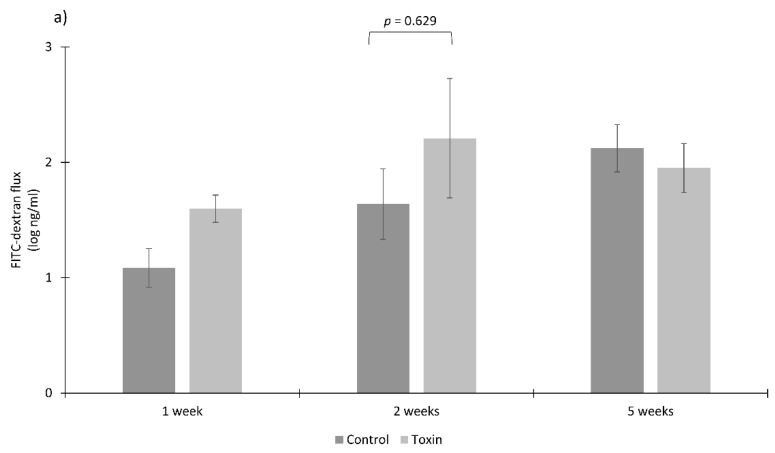

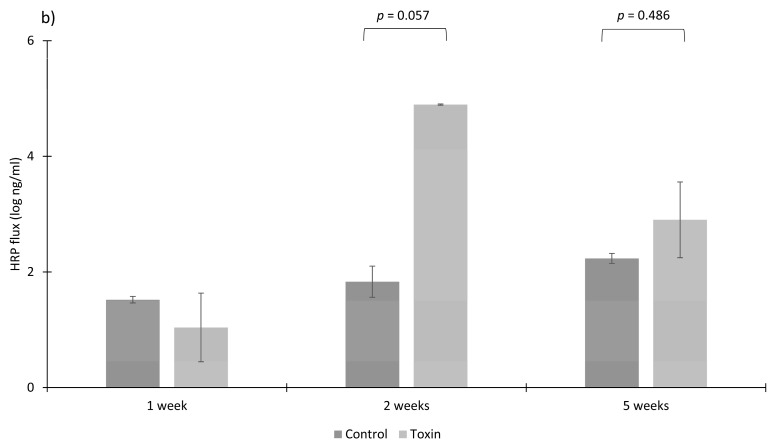
Permeability to macromolecules FITC-dextran (**a**) and HRP (**b**) of colon epithelia extracted from the gut of one-, two- and five-week-old healthy piglets (*n* = 4 per age group) and treated with *C. difficile* toxins in Ussing chambers (three chambers/piglet/treatment). The Mann–Whitney U test was used to test differences between the groups (significance *p* ≤ 0.05).

Colon tissues of two-week-old piglets showed a tendency towards increased paracellular permeability to HRP (*p* = 0.057) but no difference was observed for permeability to FITC-dextran (*p* = 0.629) when treated with the toxins, as compared to non-treated tissues (Figure 5a,b).

In colon tissues from five-week-old piglets, the toxins only slightly increased permeability to HRP vs. the control (*p* = 0.486). Data obtained for the flux of FITC-dextran from the colon of one- and five-week-old piglets and for HRP from the colon of one-week-old piglets were excluded from statistical analysis due to abnormal dye concentration at the serosal side of the tissues, which finally resulted in low number of reliable replicates (*n <* 3). Such an abnormal flux of the markers could be caused by micro-perforations during preparations of colon tissues or during the incubation in Ussing chambers due to their fragility at a young age.

**Figure 6:**
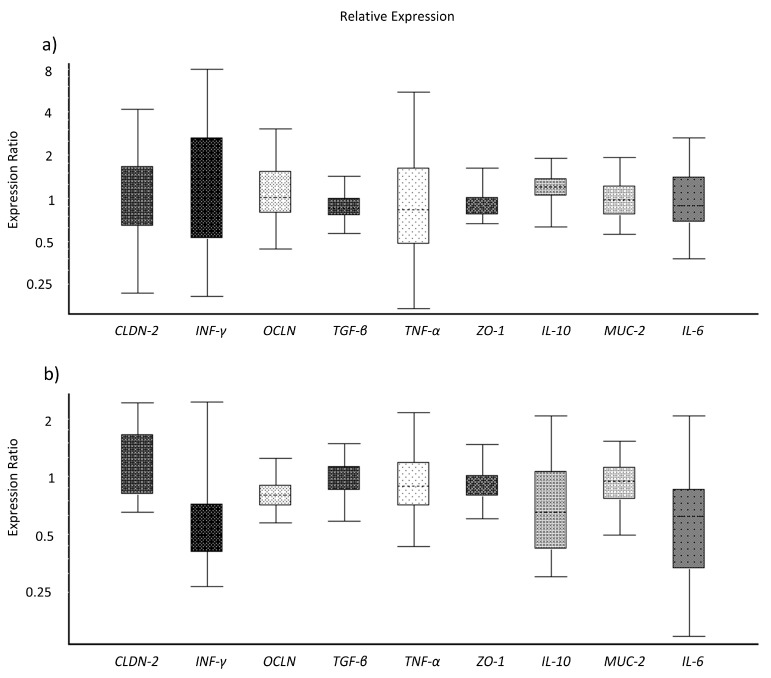
Gene expression ratios (Toxin/Control) of tight junction proteins and immune markers in the colonic tissue extracted from the gut of one- (**a**) and five-week-old (**b**) healthy piglets (*n* = 4 per age group) and treated with *C. difficile* toxins in Ussing chambers (three chambers/piglet/treatment). The gene expression data were analysed by REST software.

Toxins also downregulated the gene expression of *OCLN* in colon tissues of five-week-old piglets (*p* = 0.05) and tended to do so for *IFN-γ* (*p* = 0.072) (Figure 6a,b). The RNA extracts from two-week-old piglet colon tissues were excluded from gene expression analysis due to low RNA quality, as verified by the Bioanalyzer method.

## 4. Discussion

In this study, we show that the colon epithelium of healthy suckling and weaned piglets and IPEC-J2 cells treated with porcine colostrum are at least for a certain time period protected from *C. difficile* toxin-induced effects. The beneficial impact of mother’s milk on offspring development is unquestionable. Specifically, porcine milk is the sole source of nutrients and immune cells for the new-born piglet. In addition, it is rich in bioactive compounds, such as growth factors, microbial antigens and antibodies [24,25], which may positively influence the intestinal barrier function in piglets [14,26]. Here, the toxins showed only a slight detrimental effect on the permeability of colon tissue explants from suckling and weaned piglets. Gene expression analysis in the colon explants from five-week-old weaned piglets revealed a down regulation of *OCLN* and a trend for downregulation of *IFN-γ* in tissues treated with the toxins. Occludin together with claudins are the main components of TJ proteins and therefore play an important role in tissue stability and barrier function [27]. Occludin may be linked to the cytoskeletal F-actin through ZO-1 proteins. The dissociation of OCLN has been previously linked with the *C. difficile* toxin effect on human cell lines in vitro, being the targets for toxins [4]. Furthermore, *C. difficile* toxins have been reported to mediate acute inflammation by induction of microtubule depolymerisation in human colonocytes and induce a pro-inflammatory response in an experimental piglet model [7,28]. We have recently demonstrated that neonatal piglets fed a formula milk develop gut symptoms similar to that observed in CDI piglets and they also show lower expression of TJ proteins, as compared to suckling piglets [8]. Moreover, neonatal piglets that are switched to formula feeding are known to develop gut microbial dysbiosis, which may predispose them to CDI [8,29]. Our findings as well as previous data clearly support the importance of porcine milk including colostrum in offspring nutrition as a gut barrier for protection against CDI.

However, the piglet gut is a complex organ and certain modes of action are difficult to assess ex vivo. On the other hand, cell line models offer a possibility to study exposure effects in relation to a certain cell type. We therefore used the porcine intestinal cell model IPEC-J2 (originally isolated from neonatal piglet), which most closely resembles the intestinal pig epithelium, to study the impact of toxins on the cell integrity and viability. Although *C. difficile* toxins are reported to target colon tissues in piglets, we found that IPEC-J2 cells, which are of jejunal origin, are also susceptible to the toxins [8,30]. In addition, we combined the porcine colostrum together with *C. difficile* toxins to study whether the porcine colostrum would show any evidence of protection from cell intoxication. Here, the toxins drastically reduced IPEC-J2 cell integrity, similar to previous observations [5]. In addition, the presence of toxins led to a slightly lower gene expression ratios of TJ proteins, immune markers and finally to cell death. The reason for the only slight differences in the gene expression ratios in toxin-treated cells could be due to an early removal of the cells for further measurements, which after a two-hour incubation time from the addition of toxins to a sample collection may have not been sufficient to detect significant differences in the expression of certain genes. It is known that gene expression may differ depending on the cell lines used, time of incubation and concentration of stressors, among others [31]. However, the retrieval of the cells after a two-hour incubation time was sufficient to assess the cells by means of IF. Here, we observed drastic detrimental changes in the F-actin cytoskeleton and ZO-1 when the cells were incubated with the toxins. Similar findings on the impact of *C. difficile* toxins on human intestinal cell structure and integrity in vitro have been demonstrated before [4,6,7]. Our data support the mode of action of *C. difficile* toxins on porcine cells in a similar fashion to human cells, in which the toxins disrupt the F-actin cytoskeleton and ZO-1, the anchoring protein of other TJ proteins to the actin cytoskeleton. In contrast, the addition of porcine colostrum to the cells slightly increased the TEER values during the first five hours of incubation, supporting the beneficial role of porcine colostrum on intestinal epithelium. Neither the F-actin nor ZO-1 were negatively affected by the presence of porcine colostrum. The observed reduction in the TEER values in cells treated with porcine colostrum after 24 h of incubation could be caused by microbial growth, which is known to occur in the porcine milk [32]. To our great surprise, the porcine colostrum combined with the toxins was able to reduce the decrease in TEER values in a time-dependent manner. After two hours of incubation, the integrity of the cells treated with the porcine colostrum and toxin complex was similar to the cells treated with the porcine colostrum or control medium. Following the incubation period, the integrity of the cells treated with the porcine colostrum and toxin complex decreased; however, it remained higher compared to the cells treated with the toxins only. In addition, the IF analysis of the cells treated with the porcine colostrum and toxin complex clearly showed that the F-actin cytoskeleton and ZO-1 TJ proteins remained mostly intact. The above findings clearly support the protective role of porcine colostrum against cell intoxication caused by *C. difficile* toxins. In addition to a nourishing function of mammalian milk, it has been demonstrated that human milk oligosaccharides adhere to *C. difficile* toxins and slightly inactivate their toxicity in a cell culture [33]. It is also possible that the presence of maternal antibodies from porcine colostrum against *C. difficile* toxins A and B and a binary toxin contributed to the observed effects, as seen in our study. We have previously found IgG antibodies against *C. difficile* toxin A in these same porcine colostrum samples that were used in the present work with IPEC-J2 cells, and these were also found in the serum of the studied sows [5]. The protective role of IgG anti-toxin antibodies has been investigated before. Gnotobiotic piglets infected with toxigenic *C. difficile* followed by the administration of human monoclonal IgG anti-toxin antibodies led to a significant protection from development of systemic CDI and minimised intestinal lesions [34,35]. In addition, human monoclonal IgG anti-toxin antibodies reduced mortality from 100% to 45% in *C. difficile*-infected hamsters [36]. The potential of toxin-neutralising antibodies has also been observed in humans and currently they are commercially available for patients suffering from recurrent CDI [37]. The presence of toxin-inactivating antibodies in porcine milk and colostrum could partially explain the phenomenon of asymptomatic piglets, where *C. difficile* and its toxins are detected at high concentrations; however, piglets remain without clinical symptoms of infection [2,11,38]. In humans, for instance, asymptomatic *C. difficile* carriers are characterised by an increased serum levels of IgG anti-toxin antibodies [39]. A large proportion of immunoglobulins in porcine colostrum consists of IgG (77% on the first day of lactation), which is essential in the host defence against environmental antigens, including bacterial toxins. In contrast, IgG shows a rapid five- and 27-fold decline from the beginning to a day one and six of lactation, respectively [40]. However, piglets from the same litter usually differ in weight where the heavier and/or stronger littermates may have better access to colostrum and thus higher anti-toxin antibody uptake, which could partially support the presence of asymptomatic *C. difficile*-toxin carriers among neonatal piglets [41]. The threshold of toxin concentration in the sow needed to trigger IgG synthesis, and for how long such antibodies would circulate in the bloodstream is not known so far. Currently, sows can be vaccinated against *C. perfringens* prior to farrowing. Consequently, new-born piglets receive passive protection against *C. perfringens* toxins [42]. Although very promising, no such strategy has been developed to prevent CDI in neonatal piglets.

The immune system of neonates, including piglets, is immature and passive immunisation from their mothers is necessary until it is developed. It is recognised that the association between mother and offspring through immune and microbial programming during the neonatal period is crucial for its protection against pathogens [43,44,45]. In this context, our findings strongly support the important role of maternal protection against CDI in neonatal piglets. Although *C. difficile* outgrowth in pigs as well as in humans seems to be dependent on the microbial diversity [46,47], piglets colonised by toxigenic *C. difficile* may also be able to further develop immunity against toxins, similar to human infants [48], protecting the gut from potential intoxication and infection in the future.

## 5. Conclusions

Taken together, porcine colostrum seems to have the potential to protect the epithelial cells and colon of neonatal piglets against *C. difficile* toxin-induced effects, most probably by the presence of toxin-neutralising IgG antibodies and a nourishing role on the gut cells. Porcine colostrum may be an important determinant of immune early programming towards protection from *C. difficile* infections in offspring.
